# Valsalva retinopathy in a teenager treated with Nd:YAG laser posterior hyaloidotomy

**DOI:** 10.11604/pamj.2021.38.161.28100

**Published:** 2021-02-12

**Authors:** Omar Mahmoud Solyman, Ahmed Abdelfatah Ghalwash

**Affiliations:** 1 Research Institute of Ophthalmology, Giza, Egypt

**Keywords:** Valsalva retinopathy, subhyaloid hemorrhage, Nd:YAG laser

## Image in medicine

A 14-year-old otherwise healthy male patient presented with acute left loss of vision for 3 hours following weightlifting. Examination showed dense pre-macular sub-hyaloid hemorrhage (A). The patient and his parents did not like to wait for gradual spontaneous resolution of the hemorrhage. Neodymium-doped yttrium aluminum garnet (Nd:YAG) laser posterior hyaloidotomy was performed with quick drainage of blood into the vitreous cavity (B, C, D). He maintained stable vision and retinal exam over 20 months of follow-up. Valsalva retinopathy can affect pediatric as well as adult age groups. Nd:YAG laser posterior hyaloidotomy is minimally invasive and safe procedure that provides rapid visual rehabilitation in cooperative patients.

**Figure 1:**
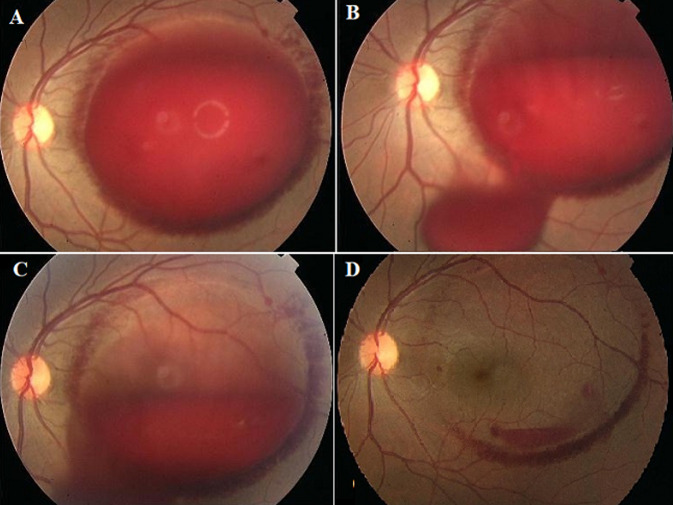
A) left elevated dome-like dense collection of pre-macular sub-hyaloid hemorrhage; B, C) quick egress of blood from posterior hyaloidotomy to the vitreous cavity one and two hours after Nd:YAG laser procedure respectively; D) near complete resolution of premacular hemorrhage on follow-up three days after procedure

